# South African Podiatry Students' Experiences of Their Clinical Learning Environment

**DOI:** 10.1002/jfa2.70092

**Published:** 2025-10-11

**Authors:** Moothee Melissa, Ntuli Simiso

**Affiliations:** ^1^ Department of Podiatry Faculty of Health Sciences University of Johannesburg Johannesburg South Africa

**Keywords:** clinical learning environment, experiences, experiential learning, podiatry, students

## Abstract

**Background:**

The role and purpose of a clinical learning environment (CLE) is to help students become confident and independent practitioners. Exposure to clinical learning environments provides podiatry students with essential experiential learning opportunities and skills that are difficult to obtain elsewhere. Anecdotal evidence suggests that podiatry students encounter various obstacles in CLEs, although these obstacles have not been thoroughly described. This article, therefore, aims to understand and describe South African podiatry students' experiences of their clinical learning environment.

**Methods:**

A qualitative research method was employed. A purposive sampling strategy was used to select undergraduate podiatry students from the University of Johannesburg to participate in a focus group discussion. Eight students took part in the focus group discussion, which was audio recorded and transcribed verbatim. Data were analysed using Giorgi's qualitative thematic analysis.

**Results:**

Three main themes were identified: (i) Attitudes of supervising clinicians, (ii) Student confidence and (iii) Shortage of supervising clinicians.

**Conclusion:**

Overall, the experiences of podiatry students in their clinical environment were negative. Understanding these obstacles may offer insight into the improvement of the clinical learning environment, and it may be possible to facilitate the development of competent and confident podiatrists.

**Abbreviation**
CLE: clinical learning environment

## 1 Background

Clinical education requires creating a supportive atmosphere for learners to be prepared for their future profession upon graduation [1, 2, 3]. A nurturing and empowering clinical learning environment prepare students to function independently [4]. Podiatry education in South Africa provides a combination of theoretical and clinical learning experiences to train students with the required knowledge, skills and attitudes for postgraduate professional practice. Podiatry education in South Africa offers a comprehensive combination of theoretical and clinical learning experiences to equip students with the necessary knowledge, skills and attitudes required for postgraduate professional practice. The University of Johannesburg is the sole institution on the African continent that provides podiatry training. Clinical learning environments comprise of a university clinic, primary health care clinics, public hospitals and frail care centres, where podiatry students are supervised by qualified and clinically experienced podiatrists. However, most supervising clinicians are full‐time clinicians employed at the clinical training sites and supervise students on a part‐time basis. Notably, these clinicians lack teaching and/or assessment experience, which may impact their capacity as clinical supervisors. The institution offers an annual clinical supervisor induction and training workshop for all supervising clinicians. Clinical education is a crucial domain in the development of the podiatry profession, enabling podiatry students to convert theoretical knowledge into practical skills and apply them to patient care. The quality of clinical exposure for students is a key factor in determining the quality of podiatry education, and it has a substantial impact on their learning outcomes in this field. A study by Ebrahim et al. highlights the key aspects influencing clinical supervision in undergraduate occupational therapy programmes, the study findings emphasise the importance of having a protocol, sequence of preparation and procedure, and training of personnel in informing quality of supervision [3].

The clinical learning environment of podiatry students involves a vast amount of time spent treating patients under the supervision of qualified podiatrists. Students should be encouraged to adopt different learning strategies that encourage active learning, coupled with the need to develop clinical reasoning skills [5, 6]. Hence, the need for an optimal clinical learning environment is key in the acquisition of clinical training. In support of the statement mentioned above, a study found that an optimal clinical learning environment has a positive impact on students' professional development [7]. However, some factors may hinder clinical learning, such as inappropriate clinical evaluation, an unsupportive learning environment and inadequate clinical supervision [7]. The exploration of nursing students' clinical supervision experiences revealed a central theme of professionalism [8]. The study identified positive aspects, including the demonstration of knowledge and positive reinforcement; negative aspects such as poor time management; unskilled and incompetent supervisors and abusive behaviour [8]. The podiatry department at the University of Johannesburg currently holds only six full‐time academic staff members who play the role of clinical supervisors. The shortage of staff is problematic and has significant constraints on clinical supervision. Upon graduation, most podiatrists go into private practice, leaving a minority to follow a career in academia. This unequal distribution of qualified podiatrists does pose issues for the undergraduate training of podiatrists.

A study to explore the experiences of nursing students during clinical practicum found that lack of respect and role models, excessive demands and hostile environment were key themes [9]. Another study explored the experiences of Iranian nursing students in the clinical learning environment, highlighted the lack of consistency in teaching; evaluation; limited learning opportunities, and inappropriate communication and interaction [10]. This study was the first study to explore the challenges faced by South African podiatry students in their clinical learning environment. Apart from a study by Ntuli et al. which dealt with podiatry students' perceptions of feedback given as part of clinical training. This study highlighted that the majority (86%) of students indicated they would prefer to receive feedback in private [1]. The attitudes of clinical supervisors and personal motivation have been identified as the key themes that emerge from studies which explored students' perception of challenging learning environments [11]. Clinical supervision is essential to podiatry students' transformation into skilled podiatrists in. Effective communication, emotional intelligence, cultural sensitivity, collaboration and conflict resolution are some of the skills that professionals need to demonstrate as part of professionalism [8]. The key environment for student podiatrists' growth and development into competent professionals is the clinical learning environment, where they receive their training and gain practical experience. Understanding the obstacles podiatry students face in their CLE could allow for a holistic consideration of the needs of students and allows for the improvement thereof. This article aims to describe the experiences South African podiatry students face in their clinical learning environment.

## 2 Methods

### 2.1 Participants

The target population for the study consisted of all registered podiatry students in their second, third or fourth years of study, provided they were over the age of 18 years. Eligible participants were selected based on their extent of exposure to a clinical learning environment (CLE), at the University of Johannesburg Podiatry clinic. This university clinic serves primarily as a training site for undergraduate podiatry students and is supervised by internal clinicians/academics employed by the university.

A total of eight students participated in the focus group discussion. The selection process involved two students in their second year of study and three students from the third and fourth years. The extent of clinical exposure varies among students in different years of study. First‐year students were excluded from the study due to their limited patient exposure, as they do not yet have clinical placement as part of their training. The focus group participants consisted of two students in their second year of study and three students from the third and fourth years. It is worth noting that the extent of clinical exposure is significantly higher for third and fourth‐year students compared to those in their second year. As a result, second‐year students are considered junior students, while third and fourth‐year students are regarded as senior students.

### 2.2 Materials

For this qualitative methods study, a focus group discussion was selected as the most suitable approach to gain an in‐depth understanding of podiatry student experiences within their clinical learning environment. Purposive sampling, consistent with the study design of Green and Thorogood, was employed in this study [12]. Volunteering participants signed an informed consent form, which allowed them to participate in the study and be audio recorded. To protect participant confidentiality, no individual identifying data was recorded. Participants were given name tags with numbers one through eight. The focus group discussion was guided by the question, ‘What are the challenges you face during your clinical learning environment?’ Each discussion lasted between 40 and 60 min.

### 2.3 Procedures

Ethical clearance from the Faculty of Health Sciences research ethics committee at the University of Johannesburg (REC‐2160‐2023) was granted for this study. Before the focus group discussion began, participants were informed of the study's purpose and reminded that they had the right to withdraw from the study at any time without facing any repercussions. To maintain confidentiality and ensure anonymity, all personal information that could potentially reveal participants' identities was not captured.

### 2.4 Data Analysis

Thematic analysis was applied by the researcher following the framework outlined by Giorgi (2009) [13]. Coding of the transcript was conducted using ATLAS.ti, a software designed for qualitative analysis. The researcher (M.M.) then collated and clustered the codes into potential themes and subthemes, which were subsequently reviewed and validated by the researcher. The accuracy of the codes was also checked by the research supervisor (S.N.). Following this process, both authors (M.M. and S.N.) were involved in the final analysis and selection of extracts for this publication. To establish trustworthiness in the study, Lincoln and Guba's five criteria were employed [14].

## 3 Results

The focus group comprised eight participants. Table 1 outlines the study's main emerging themes, accompanied by exemplar statements. The findings were dispersed across three primary challenges identified by podiatry students: the attitude of clinical supervisors, student confidence and the scarcity of clinical supervisors.

**TABLE 1:** Main emerging themes and accompanying statements made by participants.

Theme	Statement/s
1. Attitude of clinical supervisors	*‘The clinician who was supervising me, went to an extent of inviting their colleague to embarrass me in front of my patient.’’* *‘I couldn't remember the name of one instrument I had used to treat the patient, and I got shouted at.’* *‘There isn't a space for you to think and be creative and have freewill’.* *‘lf ever you have a different opinion, then I don't think they handle that well’.* *‘We are not given that opportunity to provide our rationale.’*
2. Shortage of clinical supervisors	*‘There is a shortage of supervising clinicians at our clinics, I often have to wait for a clinician to be attended too’.* *‘When clinics are busy, we wait for a clinician to attend your case, I waited 20 min, and this delayed my patient's consultation time’.*
3. Student confidence	*‘The clinician will critique all the bad things that you've done, and you don't know how good you are in the categories you are excelling in, so you don't know. The only learning experience you get is what you should not have done. But what about the things that I did that are good’.* *‘Clinician provides feedback in the presence of the patient and also students who were observing the consultation and this has a negative impact on the student's confidence’.* *‘Some clinicians are guilty of belittling, and they constantly remind you that you are a student’. opinion, then I don't think they handle that well.’*

Theme one provides insight into students' perceptions of their clinical supervisors' attitudes, revealing a predominantly negative outlook. This negative attitude is reported by students and can significantly undermine the effectiveness of clinical education, ultimately having detrimental effects on students.

Theme two highlights a significant concern in the clinical setting, specifically a shortage of clinical supervisors within the podiatry department.

Theme three reflects the bruised confidence of students, leaving them feeling unsupported, isolated, and overwhelmed. This potentially has a negative impact on their well‐being and academic performance.

## 4 Discussion

The findings of this study suggest that students have concerns about their clinical learning environment. Key challenges identified include the attitude and shortage of clinical supervisors, as well as students' confidence. Students reported difficulties they experience in a clinical setting and how these obstacles impact their learning and progression.

Despite these issues, students recognise the value of guidance from clinical supervisors, which is crucial for their development in clinical practice. One student reflected, ‘*I appreciate the guidance provided by the supervisor, but an encouraging attitude would create a better learning environment.’* This sentiment is supported by a study that mentions positive attitudes among clinical staff significantly enhance students' learning experiences [5]. This study also highlighted a concerning theme: supervising clinicians with poor attitudes that negatively impact students' clinical learning environments. One student noted, *‘I always get critiqued on all the areas where I've gone wrong, and I am never praised for the areas I have excelled in.’* It is essential for clinicians to serve as role models for students and to demonstrate a caring and passionate attitude within the clinical learning environment [15]. In a clinical learning environment, teaching objectives are essential to guide the learning process while focusing on student‐centred learning and the development of professional competencies and clinical skills. These objectives should be clear to both student and supervising clinician, promoting active student involvement and facilitating the development of higher‐order thinking, clinical reasoning and decision‐making skills required for competent, safe practice. Supervisors may not fully comprehend their role as mentors, which involves offering support to students during their clinical placements. This lack of clarity can lead to students perceiving their supervisors as having an uncaring approach to supervision. Clinical supervisors may have high expectations that could potentially hinder their ability to relate to a student's level of thinking. The reasons behind clinical supervisors' poor attitudes are currently unknown and warrant further investigation.

Clinical practice is a complex and dynamic process through which students gain experience and translate theoretical knowledge into practical applications in a clinical setting [16, 17]. Practice‐based teaching is designed to provide students with a solid foundation in scientific knowledge and facilitate its practical application within a clinical learning environment. In the undergraduate training of podiatry in South Africa, a significant portion of the course is dedicated to practice‐based teaching in a clinical setting. This emphasis is justified, as clinical practice is a crucial component of podiatry education, effectively bridging the gap between theoretical knowledge and practical skills. However, in understaffed clinical settings, supervision may not be prioritized, with supervisors often feeling pressured to focus on direct patient care, leaving them with limited time and energy to devote to student guidance. This can lead to a rushed, unsupportive or even dismissive approach to supervision. The podiatry department at the University of Johannesburg faces staff shortages, which have a negative impact on clinical supervision. This is because supervisors often have conflicting demands between teaching and clinical responsibilities. When these demands are not adequately balanced, the quality of supervision can decline.

The clinical learning environment encompasses a psychological combination of cognitive, cultural, social, mental, emotional, educational and motivational factors that influence the interaction between clinical supervisors and students [11]. The clinical learning environment is a critical aspect of the quality and effectiveness of clinical education. It plays a significant role in teaching and learning and can have a profound impact on the learning processes of students. The clinical learning environment influences academic performance, motivation, and psychological well‐being, making it a crucial determinant of students' behaviour [18, 19].

This study aligns with previous findings in other medical fields, which underscores the importance of a constructive clinical learning environment for facilitating a positive learning experience [20]. A previous study highlights numerous benefits that clinical learning environments provide, such as the development of clinical knowledge, professional socialisation and enhancement of confidence, among others [21]. However, they also note that these environments can be challenging, unpredictable and stressful. Positive clinical learning environments are associated with favourable outcomes, whereas negative environments can result in poor learning experiences [21]. Achieving optimal learning performance is difficult without a supportive environment. Thus, understanding the learning environment is essential for the development of effective curricula [22].

## 5 Conclusion

Clinical exposure is a fundamental aspect of clinical teaching and learning. It helps identify areas for student development and reinforces good practices, while also motivating students to achieve their learning outcomes. This study highlights the challenges faced by podiatry students in the clinical learning environment. There is a clear need for improvement in this environment. Recommendations include promoting a positive atmosphere and teamwork, enhancing interaction skills among podiatry students and increasing learning opportunities within the clinical setting. Addressing these negative attitudes requires a multi‐faceted approach, including robust training programs for supervisors, fostering a supportive organisational culture, managing workload effectively and promoting open communication channels.

## Author Contributions

**Moothee Melissa:** conceptualization, writing – original draft, writing – reviewing and editing. **Ntuli Simiso:** conceptualization, writing – reviewing and editing.

## Ethics Statement

The study was approved by the Faculty of Health Sciences research ethics committee at the University of Johannesburg (REC‐2160‐2023). Written consent was obtained from all participants before taking part.

## Consent

The authors have nothing to report.

## Conflicts of Interest

The authors declare no conflicts of interest.

## Data Availability

The datasets used and/or analysed in the current study are available from the corresponding author upon reasonable request.
